# Reliable Object Pose Alignment in Mixed-Reality Environments Using Background-Referenced 3D Reconstruction

**DOI:** 10.3390/s26082453

**Published:** 2026-04-16

**Authors:** Gyu-Bin Shin, Bok-Deuk Song, Vladimirov Blagovest Iordanov, Sangjoon Park, Soyeon Lee, Suk-Ho Lee

**Affiliations:** 1 HCI Laboratory, Kookmin University, Seoul 02707, Republic of Korea; sgb1027@kookmin.ac.kr; 2 Hyper-Reality Metaverse Research Laboratory, ETRI (Electronics and Telecommunications Research Institute), Daejeon 34129, Republic of Korea; bdsong@etri.re.kr; 3 Warrior Augmentation Research Section, ETRI (Electronics and Telecommunications Research Institute), Daejeon 34129, Republic of Korea; vladimirov@etri.re.kr (V.B.I.); sangjoon@etri.re.kr (S.P.); sylee@etri.re.kr (S.L.); 4 Department Computer Engineering, Dongseo University, Busan 47011, Republic of Korea

**Keywords:** 3D reconstruction, camera sensors, virtual reality, human–computer interaction, 3D pose estimation

## Abstract

Accurate alignment of real-world object poses with their virtual counterparts using sensors, e.g. cameras, is essential for consistent interaction in mixed-reality systems. However, objects can undergo abrupt, untracked movements during periods when a tracking system is inactive, e.g., overnight, causing stored pose records to become inconsistent with the real scene and breaking user interaction in the virtual environment. Off-the-shelf 3D reconstruction networks such as MASt3R (Matching and Stereo 3D Reconstruction) method provide metrically scaled 3D point maps and pixel correspondences, but they are trained on static scenes and therefore fail to produce reliable object correspondences when the object has moved. We propose a robust pipeline that combines MASt3R’s metrically scaled 3D outputs with a background-based alignment strategy to recover and apply the true pose change of moved objects. Our method first segments foreground and background and extracts 3D background point sets for a reference day and a current day. An affine transformation between these background point sets is estimated via a standard registration technique and used to express the current-day object 3D coordinates in the reference coordinate frame. Within that unified frame we compute the object pose change and apply the resulting transform to the virtual object, restoring real–virtual consistency. Experiments on real scenes demonstrate that the proposed approach reliably corrects pose misalignments introduced during inactive periods and substantially improves over applying MASt3R alone, thereby enabling restored and consistent user interaction in the virtual environment.

## 1. Introduction

In the field of mixed-reality systems using sensors like cameras, accurate alignment of real-world object poses with their virtual counterparts is a fundamental requirement for consistent interaction. Misalignments directly degrade user immersion and can disrupt critical applications such as digital twins, industrial inspection, and interactive AR (Augmented Reality)/VR (Virtual Reality) experiences. However, ensuring reliable alignment over extended periods remains difficult, particularly when objects undergo abrupt, untracked movements during inactive intervals of a tracking system, for example, during overnight. Such events cause stored pose records to diverge from the real scene, breaking the consistency between physical and virtual environments. Although recent advances in deep learning and 3D reconstruction have produced powerful off-the-shelf networks such as MASt3R (Matching and Stereo 3D Reconstruction) method [1], which generate metrically scaled 3D point maps and pixel correspondences, these models are trained primarily on static scenes. Consequently, they fail to produce reliable object correspondences once the object has been displaced or reoriented. This limitation motivates the need for methods that not only reconstruct static object geometry but also recover true pose changes in dynamic or intermittently tracked environments.

To address this problem, we propose a robust pipeline that integrates MASt3R’s metrically scaled 3D outputs with a background-based alignment strategy to restore real–virtual consistency. Specifically, our method first segments foreground and background and extracts 3D background point sets for a reference day and a current day. An affine transformation between the background point sets is then estimated using standard point cloud registration techniques, enabling the current-day reconstruction to be expressed in the reference coordinate frame. Within this unified frame, the object’s pose change can be reliably computed and applied to the virtual object, restoring alignment without requiring continuous tracking or prior motion information.

The proposed method distinguishes itself from existing multi-view registration and background-based alignment approaches in two key aspects: First, the proposed method recovers object pose changes purely from sparse multi-view images before and after motion, without requiring temporal continuity or tracking history. Most existing approaches, for example, SLAM (Simultaneous Localization and Mapping) and tracking-based AR systems, depend on continuous sensing, or prior motion models. In contrast, the proposed method recovers object pose changes purely from sparse multi-view images before and after motion, without requiring temporal continuity or tracking history. Second, the proposed method can handle abrupt and untracked changes in camera viewpoints, which is primarily enabled by the use of the MASt3R framework within our pipeline. However, unlike the original MASt3R method—which is designed to handle temporal view variations under the assumption of static scene geometry—our approach addresses the complementary and more challenging scenario in which the scene geometry itself changes due to object motion. As a result, the proposed method enables reliable pose recovery in the presence of object motion, temporal discontinuity, and abrupt changes in the poses of both the background and the object.

We evaluate the proposed approach through qualitative and quantitative experiments on real-world scenes, demonstrating that it reliably corrects pose misalignments introduced during inactive periods. The results show substantial improvements over directly applying MASt3R alone, particularly under large object displacements and rotations. In summary, this work makes the following contributions: (i) it identifies and formalizes the problem of pose inconsistency caused by intermittent tracking in MR (Mixed Reality) systems; (ii) it introduces a background-aligned pose recovery pipeline that complements learning-based 3D reconstruction; and (iii) it demonstrates improved robustness and consistency in real–virtual alignment for long-term MR deployments. Together, these contributions enable more persistent and trustworthy MR experiences. The remainder of this paper is organized as follows. Section 2 reviews existing works on pose estimation and introduces the MASt3R method that forms the basis of the proposed approach. Section 3 details the proposed method. Section 4 presents the experimental results, and Section 5 concludes the paper.

## 2. Related Work

### 2.1. Object Pose Estimation

Three-dimensional object pose estimation from monocular videos obtained by camera sensors has become an active research field with applications in augmented and mixed reality, robotics, and human–computer interaction. Unlike traditional 2D detection, 3D pose estimation requires resolving depth ambiguity, occlusion, and domain variability, making the task fundamentally ill-posed. The first generation of methods adopted 2D-to-3D lifting networks, where detected 2D keypoints were directly mapped to 3D coordinates through multi-layer perceptrons [2]. Although simple, these approaches lacked robustness against noisy detections and failed to incorporate temporal dependencies.

To address this, methods such as VideoPose3D [3] integrated temporal convolutions, improving continuity across sequential frames. Another important direction relied on multi-view geometry with datasets such as Human3.6M and MPI-INF-3DHP [4]. These methods achieved high accuracy but were constrained by the high cost and complexity of multi-camera setups. With the rapid development of deep learning, transformer architectures became dominant in the monocular pose estimation field. PoseFormer [5] introduced spatio-temporal attention mechanisms, while KTPFormer [6] and MotionAGFormer [7] combined kinematic priors and graph convolutional networks (GCNs) to enforce structural consistency. These designs improved robustness to occlusion and viewpoint changes, and lightweight transformer variants enabled near real-time deployment for AR/VR and robotics.

Generalization to real-world scenarios has also been a focus. JoyPose [8] employed evolutionary augmentation with anatomy-aware learning, while FreeMan [9] proposed a benchmark dataset reflecting natural environments with clutter and lighting variability. Self-supervised and domain adaptation techniques [10,11] further reduced reliance on large-scale annotated datasets, enabling deployment in diverse conditions. In addition, recent efforts have addressed robustness under challenging sensing conditions. For instance, the work in [12] proposes a hybrid camera pose estimation strategy that improves alignment robustness by combining semi-automatic alignment with ICP-based refinement. Furthermore, in [13], a high-quality space target dataset incorporating extreme illumination characteristics was introduced to address the challenges of 3D reconstruction under severe lighting variations.

While monocular pose estimation methods primarily assume continuous tracking, they cannot inherently resolve pose drift or untracked movements. To address this limitation, research has explored integrating 3D reconstruction and dense matching. In particular, MASt3R [1] extends DUSt3R (Dense Unconstrained Stereo 3D Reconstruction) by producing metrically scaled point maps and dense pixel correspondences, enabling 3D-aware matching rather than relying solely on 2D features. The versatility of MASt3R has inspired several extensions. MASt3R-SLAM [14] incorporates MASt3R into a dense monocular SLAM framework, achieving incremental mapping, loop closure, and global optimization in real time. Optimized versions such as Speedy MASt3R [15] improve inference efficiency through fast nearest-neighbor search and mixed-precision inference. In addition, semantic extensions such as SAB3R [16] and human-centric HAMSt3R [17] integrate scene semantics, segmentation, and human–environment reconstruction, expanding applicability to interactive environments. Despite these advances, evaluations on complex photogrammetric datasets indicate that while MASt3R outperforms traditional SfM under sparse views, it still struggles in highly cluttered or large-scale environments [18].

### 2.2. The MASt3R Network

The technique proposed in this paper relies heavily on the MASt3R (Matching and Stereo 3D Reconstruction) network [1], recently introduced by the Naver Europe team as a breakthrough framework for 3D reconstruction using images or videos obtained by camera sensors. MASt3R takes as input two images of the same scene captured from different viewpoints and outputs 3D point maps for both images, represented in the camera coordinate frame of the first image. That is for images $I^{1}$ and $I^{2}$ of resolution W×H, the output becomes $X^{1,1}\in R^{W\times H\times 3}$ and $X^{1,2}\in R^{W\times H\times 3}$, respectively, where $X^{n,m}$ represents the 3D pointmap corresponding to $I^{m}$ but with respect to the camera coordinate frame of *n*-th image:

(1) $$X^{1,1},X^{1,2}=MASt3R\left(I^{1},I^{2}\right).$$

Here, the 3D pointmap X defines a one-to-one correspondence between image pixels $I_{i,j}$ and 3D scene points $X_{i,j}$, where (i,j) denotes the pixel coordinates. In addition to generating 3D point maps, MASt3R also provides pixel correspondences between the two input images. Furthermore, all 3D coordinates are metrically scaled, enabling accurate depth perception and spatial understanding. However, MASt3R performs effectively only when the objects in the scene are static. Therefore, it cannot be directly applied to our problem. To address this limitation, it is essential to employ an additional method in conjunction with MASt3R.

## 3. Proposed Method

### 3.1. Problem Statement

**Figure 1:**
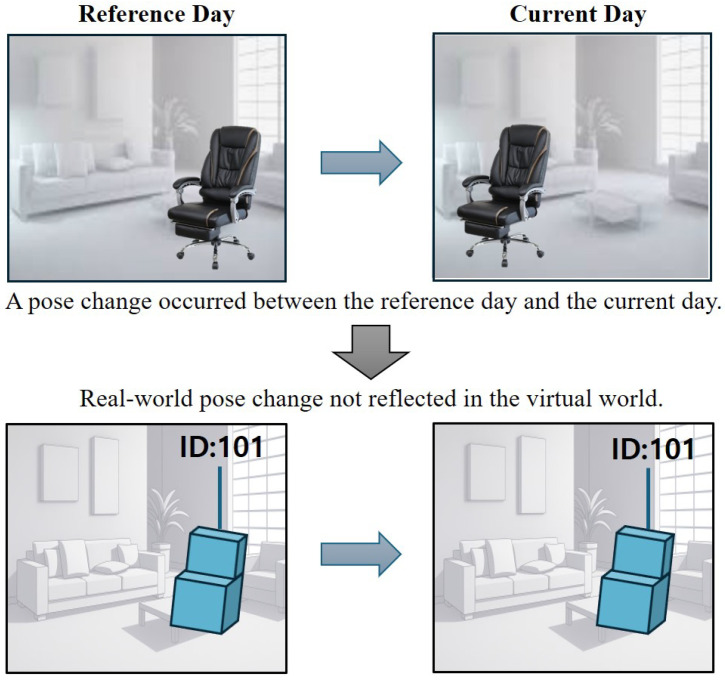
Conceptual illustration of the problem statement. The upper row shows images captured in the real world, while the lower row shows the corresponding virtual environment. An abrupt pose change occurred in the object of interest during the inactive period of the tracking system. As a result, the virtual object’s pose no longer matches its real-world counterpart, and user interactions with the real-world object cannot be properly reflected in the virtual world.

Figure 1 illustrates the concept of the problem addressed in this study. The objective is to align the pose of the object of interest in the real world with its counterpart in the virtual world. In other words, the real-world object’s pose must be accurately reflected in the virtual environment. Without proper pose alignment, the interactions with the object observed in the real world cannot be applied in the virtual world.

However, a problem arises when the pose of objects in the real world changes during the inactive period of the tracking system, which is responsible for monitoring pose changes in real time and applying them to the corresponding objects in the virtual world. For instance, during the night, when the tracking system is inactive, a cleaning staff member may inadvertently move the object of interest while cleaning. As a result, even though the object’s pose with a specific ID has been stored in the database, the pose information loaded the next day no longer reflects the object’s actual position. Consequently, the user can no longer properly interact with the corresponding virtual object.

**Figure 2:**
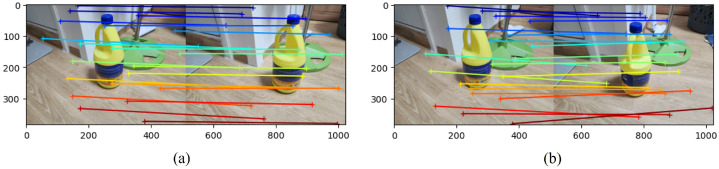
Illustration showing that correspondence estimation becomes inaccurate when the object moves. (**a**) Estimated correspondences when the object remains stationary. (**b**) Estimated correspondences when the object has moved.

The direct application of the MASt3R method cannot resolve the problem for two main reasons. First, when the images from the reference day and the current day are input into the MASt3R network, the method fails to accurately estimate the correspondences between matching pixels on the object. This issue is illustrated in Figure 2. It can be observed that when the object is in motion, the correspondences between its pixels are not accurately established. This limitation primarily arises because MASt3R is trained on scenes with static backgrounds and objects. Consequently, it fails to accurately estimate correspondences when the object has moved. Therefore, a more sophisticated approach is required to address this problem. To address this, we assume that after completing interactions with the digital twin, the user captures images of the scene. Before resuming interaction the following day, the user captures another set of images. Based on these images, the digital twin system can update the positions of moved objects and adjust the poses of objects whose configurations have changed. Within this usage scenario, users are likely to capture images under relatively controlled conditions—such as when no people are present or when objects are static—thereby reducing the difficulties with dynamic backgrounds. If dynamic environments need to be considered, additional modules (e.g., dynamic background filtering) can be incorporated into our current framework to handle such cases.

### 3.2. Overview of the Proposed Method

**Figure 3:**
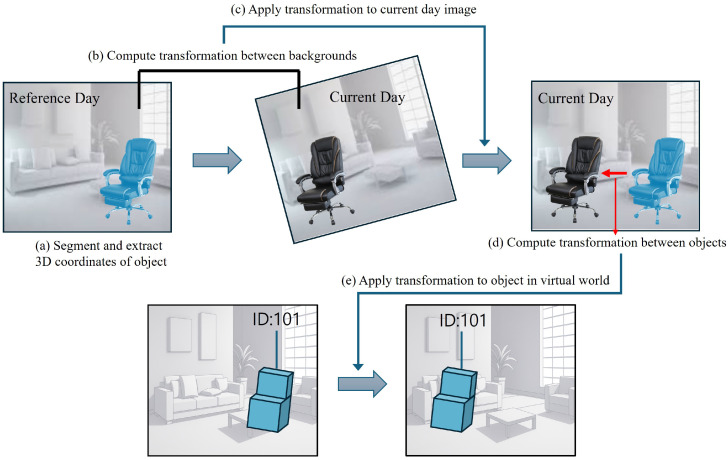
**Overview of the proposed method**. (**a**) The 3D coordinates of the object are extracted. (**b**) The transformation between the background 3D coordinates of the reference day and the current day is estimated. (**c**) The 3D coordinates of the object on the current day are transformed into the coordinate system of the reference day. (**d**) The pose change between the moved object and the initial object is estimated. (**e**) The estimated pose change is applied to the virtual object in the virtual environment.

Figure 3 presents an overview of the proposed method. First, the 3D coordinates of the object are extracted with respect to the coordinate system of the reference day (Section 3.3). Then, the 3D background coordinates of the reference day are obtained from two images captured on that day, and likewise, the 3D background coordinates of the current day are derived from two images captured on the current day. To ensure that only background information is used, segmentation methods are employed to separate the foreground (object) region from the background. Next, the affine transformation between the 3D background coordinates of the reference and current days is estimated (Section 3.4). Using this transformation, the 3D object coordinates of the current day are converted into the coordinate system of the reference day (Section 3.5). Finally, within this unified coordinate frame, the transformation between the object poses of the reference and current days is computed and applied to the virtual environment, aligning the virtual object with its real-world counterpart as it changes from the reference pose to the current pose (Section 3.6).

The following notations are used throughout this paper: $I^{k,m}$ refers to the *m*-th image obtained on the *k*-th day where the *m*-th image is obtained by the camera at the *m*-th viewpoint, where m=1,2, and $X^{l,k,m}$ refers to the pointmap of $I^{k,m}$ with respect to the *l*-th coordinate system.

### 3.3. Initial 3D Object Reconstruction Using Multi-View Images (MASt3R)

The goal of this section is to obtain the object’s 3D coordinates(3D pointmap) expressed in the coordinate frame of the first viewpoint on the reference day. On the reference day, two images with different viewpoints are captured: $I^{1,1}$ and $I^{1,2}$. These images are provided as input to the MASt3R network, which outputs the corresponding pointmaps in the coordinate frame of the first camera:

(2) $$X^{1,1,1},X^{1,1,2}=MASt3R\left(I^{1,1},I^{1,2}\right).$$

Next, we extract the pointmap corresponding to the object of interest. This is achieved by applying an object segmentation method to the images, isolating the object region, and retaining only the 3D coordinates associated with this region in the pointmap. We denote this operation as ObjSeg(·), and represent the segmented 3D coordinates of the object in the first and second views as $X_{obj}^{1,1,1}$ and $X_{obj}^{1,1,2}$, respectively:

(3) $$X_{obj}^{1,1,1},X_{obj}^{1,1,2}=ObjSeg\left(X^{1,1,1},X^{1,1,2}\right).$$

### 3.4. Estimating Scene Transformation Between Observation Days

Next, we aim to estimate the transformation that maps the coordinate system of the first camera viewpoint on the current day to the coordinate system of the reference viewpoint. To achieve this, we first apply the MASt3R network to the image pair $I^{1,1}$ and $I^{2,1}$ yielding the pointmaps $X^{1,1,1}$ and $X^{1,2,1}$.

(4) $$X^{1,1,1},X^{1,2,1}=MASt3R\left(I^{1,1},I^{2,1}\right).$$

Similarly, we apply the MASt3R network to the image pair $I^{2,1}$ and $I^{2,2}$ obtaining the pointmaps $X^{2,2,1}$ and $X^{2,2,2}$.

(5) $$X^{2,2,1},X^{2,2,2}=MASt3R\left(I^{2,1},I^{2,2}\right).$$

Since our goal is to transform the 3D coordinates of the first image on the current day from its own camera coordinate system to that of the reference camera, we estimate the transformation between $X^{1,1,1}$ and $X^{2,2,1}$ using the background region common to both image $I^{1,1}$ and image $I^{2,1}$. That is, we first extract the 3D coordinates corresponding to the background regions of $X^{1,1,1}$ and $X^{2,2,1}$:

(6) $$X_{back}^{1,1,1},X_{back}^{2,2,1}=BackSeg\left(X^{1,1,1},X^{2,2,1}\right)$$

where BackSeg(·) refers to the operator which extract the background regions, and $X_{back}^{1,1,1}$ and $X_{back}^{2,2,1}$ are the 3D coordinates related to the background regions of $X^{1,1,1}$ and $X^{2,2,1}$, respectively. Now using the corresponding points in $X_{back}^{1,1,1}$ and $X_{back}^{2,2,1}$, we estimate the transformation—comprising scale($\sigma^{2\to 1}$), rotation($R^{2\to 1}$), and translation($t^{2\to 1}$)—that maps the 3D coordinates of the first image on the current day to those of the reference camera. This can be done by solving the following minimization problem:

(7) $$\sigma^{2\to 1},R^{2\to 1},t^{2\to 1}=\underset{\sigma ,R,t}{\arg\min}\sum_{(i,j)\in M}{∥\sigma \left(RX_{back,i}^{1,1,1}+t\right)-X_{back,j}^{2,2,1}∥}^{2}.$$

Here, *i* and *j* denote pixel positions that have mutual correspondences in $X_{back}^{1,1,1}$ and $X_{back}^{2,2,1}$, respectively. The above minimization (optimization) problem can be solved using various optimization techniques, such as the ICP (Iterative Closest Point) method [19], the Umeyama algorithm [20], or other similar approaches [21,22].

Here, M represents the set of pixel coordinates that share mutual correspondences:

(8) $$M=\left\{\left(i,j\right)|X_{back,i}^{1,1,1}=X_{back,j}^{2,2,1}\right\}.$$

### 3.5. 3D Localization of the Moved Object in the New View

The next step is to localize the position of the moved object in the new view, i.e., the view obtained after the object has been displaced. The images captured at this time are denoted as $I^{2,1}$ and $I^{2,2}$. By inputting these images into the MASt3R network, we obtain the corresponding 3D pointmaps:

$$X^{2,2,1},\;X^{2,2,2}=MASt3R\left(I^{2,1},I^{2,2}\right),$$

which are expressed in the coordinate system of the current day. Our focus here is on the 3D coordinates of the object with respect to the current day camera coordinate system. These can be extracted from $X^{2,2,1}$ or $X^{2,2,2}$ by isolating the object region. Formally, we define the extraction of the 3D foreground (object) coordinates as:

(9) $$X_{obj_{mov}}^{2,2,1}=ObjSeg\left(X^{2,2,1}\right),$$

where ObjSeg(·) denotes the operator that segments the object region from the 3D pointmap.

### 3.6. Computing the Object Displacement

Finally, to compare the relative pose of the moved object with that of the original object in the same reference coordinate system, we first transform the moved object’s coordinates into the reference frame:

(10) $$X_{obj_{mov}}^{1,2,1}=\sigma^{2\to 1}\left(R^{2\to 1}X_{obj_{mov}}^{2,2,1}+t^{2\to 1}\right),$$

where $\sigma^{2\to 1}$, $R^{2\to 1}$, and $t^{2\to 1}$ denote the scale, rotation, and translation that map the current day coordinates to the reference frame. Then, finally, we estimate the transformation between the moved object and the original object by solving the following optimization problem:

(11) $$\sigma^{obj},R^{obj},t^{obj}=\underset{\sigma ,R,t}{\arg\min}\;\sum_{\left(i,j\right)\in M_{obj}}{∥\sigma \left(RX_{obj_{mov},i}^{1,2,1}+t\right)-X_{obj,j}^{1,1,1}∥}^{2},$$

where $M_{obj}$ denotes the correspondence set between the transformed moved-object points and the original object points:

(12) $$M_{obj}=\left\{\left(i,j\right)\;|\;{X_{obj_{mov}},i}^{1,1,1}↔X_{obj,j}^{1,1,1}\right\}.$$

The parameters $\sigma^{obj},R^{obj},t^{obj}$ constitute the final transformation applied to the corresponding virtual objects to correct pose misalignment in the virtual world. Once estimated, these parameters update the scale, rotation, and translation of the virtual objects to restore consistency with their real-world counterparts. Algorithm 1 summarizes the overall workflow of the proposed method.

One important aspect to consider is that, in solving (7) and (11), our method differs fundamentally from conventional ICP, as it does not rely solely on iterative nearest-neighbor correspondence estimation. Instead, dense and geometrically consistent correspondences are directly obtained using MASt3R. Consequently, the optimization is performed over the correspondence sets defined in (8) and (12). This shifts the nature of the alignment problem: rather than jointly estimating correspondences and transformations, the proposed method solves for the rigid transformation given a set of reliable matched point pairs. As a result, the method is significantly less sensitive to initialization and partial overlap, since incorrect nearest-neighbor associations—one of the primary failure modes of ICP—are effectively avoided.

From an implementation perspective, the problems in (7) and (11) are first solved using a standard SVD-based method (i.e., Procrustes alignment), which provides a closed-form and globally optimal solution under known correspondences. To enhance robustness against outliers and partial overlap, a correspondence filtering step is applied prior to optimization. Specifically, correspondences exhibiting large reprojection errors or geometric inconsistencies are removed using a distance threshold τ (set to 0.05 m) in our implementation). Finally, the estimated transformation is further refined using a small number of iterations (10–20) of point-to-plane ICP, initialized with the closed-form solution. Since the initial estimate is already close to the optimum, ICP serves only as a local refinement step.

The overall computation time of the proposed method is primarily determined by two major components: MASt3R and the Iterative Closest Point (ICP) method. For MASt3R, given an input image of size H×W, the feature extraction stage has a computational complexity on the order of O(HW), while the dense matching stage (e.g., correlation or attention-based operations) can reach up to $O(HW^{2})$ in naive global matching. In practice, optimized implementations significantly reduce this cost. Although the theoretical complexity is high, the method is highly parallelizable on GPUs (Graphics Processing Units), enabling real-time or near real-time performance on modern hardware (typically on the order of tens to hundreds of milliseconds per image pair).
**Algorithm 1** Proposed Method for Object Pose Re-Estimation**Require:** Images $I^{k,m}$ from day k∈{1,2} and viewpoint m∈{1,2}**Ensure:** Object displacement parameters $\sigma^{obj},R^{obj},t^{obj}$1:**Step 1: Initial 3D Object Reconstruction (Reference Day)**2:Obtain pointmaps using MASt3R and extract object regions:$$X^{1,1,1},X^{1,1,2}=MASt3R\left(I^{1,1},I^{1,2}\right),\;\;\;\;\;\;X_{obj}^{1,1,1},X_{obj}^{1,1,2}=ObjSeg\left(X^{1,1,1},X^{1,1,2}\right)$$3:**Step 2: Estimating Scene Transformation (Day 1 → Day 2)**4:Apply MASt3R across days (Day 1 = Reference Day, Day 2 = Current Day):$$X^{1,1,1},X^{1,2,1}=MASt3R\left(I^{1,1},I^{2,1}\right),\;\;\;\;\;\;X^{2,2,1},X^{2,2,2}=MASt3R\left(I^{2,1},I^{2,2}\right)$$5:Extract background regions:$$X_{back}^{1,1,1},X_{back}^{2,2,1}=BackSeg\left(X^{1,1,1},X^{2,2,1}\right)$$6:Estimate transformation parameters:$$\sigma^{2\to 1},R^{2\to 1},t^{2\to 1}=\arg\min_{\sigma ,R,t}\sum_{(i,j)\in M}∥\sigma \left(RX_{back,i}^{1,1,1}+t\right)-X_{back,j}^{2,2,1}{∥}^{2}$$7:**Step 3: 3D Localization of Moved Object (Query Day)**8:Obtain moved object pointmaps:$$X^{2,2,1},X^{2,2,2}=MASt3R\left(I^{2,1},I^{2,2}\right)$$9:Extract object region:$$X_{obj_{mov}}^{2,2,1}=ObjSeg\left(X^{2,2,1}\right)$$10:**Step 4: Transforming to Reference Frame**11:Transform moved object coordinates:$$X_{obj_{mov}}^{1,2,1}=\sigma^{2\to 1}\left(R^{2\to 1}X_{obj_{mov}}^{2,2,1}+t^{2\to 1}\right)$$12:**Step 5: Computing Object Displacement**13:Estimate final displacement parameters:$$\sigma^{obj},R^{obj},t^{obj}=\arg\min_{\sigma ,R,t}\sum_{\left(i,j\right)\in M_{obj}}∥\sigma \left(RX_{obj_{mov},i}^{1,2,1}+t\right)-X_{obj,j}^{1,1,1}{∥}^{2}$$

In contrast, the computational complexity of ICP scales with both the number of points and the number of iterations. Let *N* and *M* denote the number of points in the source and target point clouds, respectively, and let *k* denote the number of iterations. When accelerated using a KD-tree, the complexity becomes O(k·NlogM). As a result, the runtime can vary significantly, ranging from milliseconds for small point clouds to several seconds (or longer) for large-scale data.

For deployment in mixed-reality applications, where users expect timely updates, the computational cost of the proposed pipeline can be further reduced through several practical optimizations. In particular, background point clouds can be downsampled prior to registration without significantly affecting accuracy, as the background primarily serves as a global reference. Additionally, correspondence sampling from MASt3R outputs can be limited to a subset of high-confidence matches to reduce computation. With these optimizations, the overall pipeline can be executed within approximately 1 s on consumer-grade hardware, making it suitable for interactive or near-interactive mixed-reality scenarios.

### 3.7. Discussion of Limitations of the Proposed Method

#### 3.7.1. Dependence on Foreground-Background Segmentation Accuracy

The proposed method relies on accurate separation of object and background regions, and its robustness is therefore inherently influenced by the quality of the employed segmentation technique. Segmentation errors may introduce spurious points into the reconstructed 3D point clouds, which can negatively affect the subsequent pose estimation. However, the proposed framework mitigates this issue in several ways. First, the strong correspondence estimation capability of MASt3R is leveraged, which is robust to partial occlusions and off-centered object views. Second, since explicit correspondences between point sets are available, outlier or falsely reconstructed points can be effectively filtered out prior to pose computation. As a result, the impact of segmentation errors on the final pose estimation is reduced.

#### 3.7.2. Challenges in Complex Background Environments

The proposed method assumes that the background scene remains static across observations acquired on different days. Under this assumption, the method can effectively handle cluttered environments, as MASt3R is known to be robust to clutter when the background remains static. This assumption is aligned with our target application scenario, in which the user captures images of the scene after completing interactions with the digital twin on a reference day, and then captures another set of images prior to resuming interaction on a subsequent day. In this context, users are naturally inclined to acquire images under relatively controlled conditions—such as when no people are present and the scene is static—thereby reducing the likelihood of background changes. However, if dynamic environments need to be considered, additional modules (e.g., dynamic background filtering) have to be incorporated into our current framework to handle such cases. Furthermore, in scenes containing multiple moving objects, re-posing must be carried out independently for each object. This necessitates a segmentation method that can accurately separate individual moving objects. Provided that such segmentation is successful, the proposed method can be applied to each object without modification. While the proposed framework can be extended to scenarios involving multiple moving objects, an additional challenge arises in preserving object identity across observations acquired on different days. In such cases, each object must be consistently associated with its corresponding instance in the reference scene. In the current framework, this can be addressed by leveraging the correspondence estimation capability of MASt3R, which enables matching between object-specific regions across views. When multiple objects are present, segmentation can be applied to separate individual object regions, after which pose estimation is performed independently for each segmented object. To maintain identity consistency, object-level features such as geometric shape, spatial location relative to the background, and appearance cues can be jointly considered when associating objects across time. We note that this approach assumes that objects are sufficiently distinguishable either geometrically or spatially. In more ambiguous cases (e.g., multiple identical objects), additional mechanisms such as instance-level descriptors or temporal consistency constraints may be required. Extending the proposed framework to robustly handle such scenarios is an important direction for future work.

#### 3.7.3. Robustness to Large Difference in the Viewpoints and Lighting Variations

**Figure 4:**
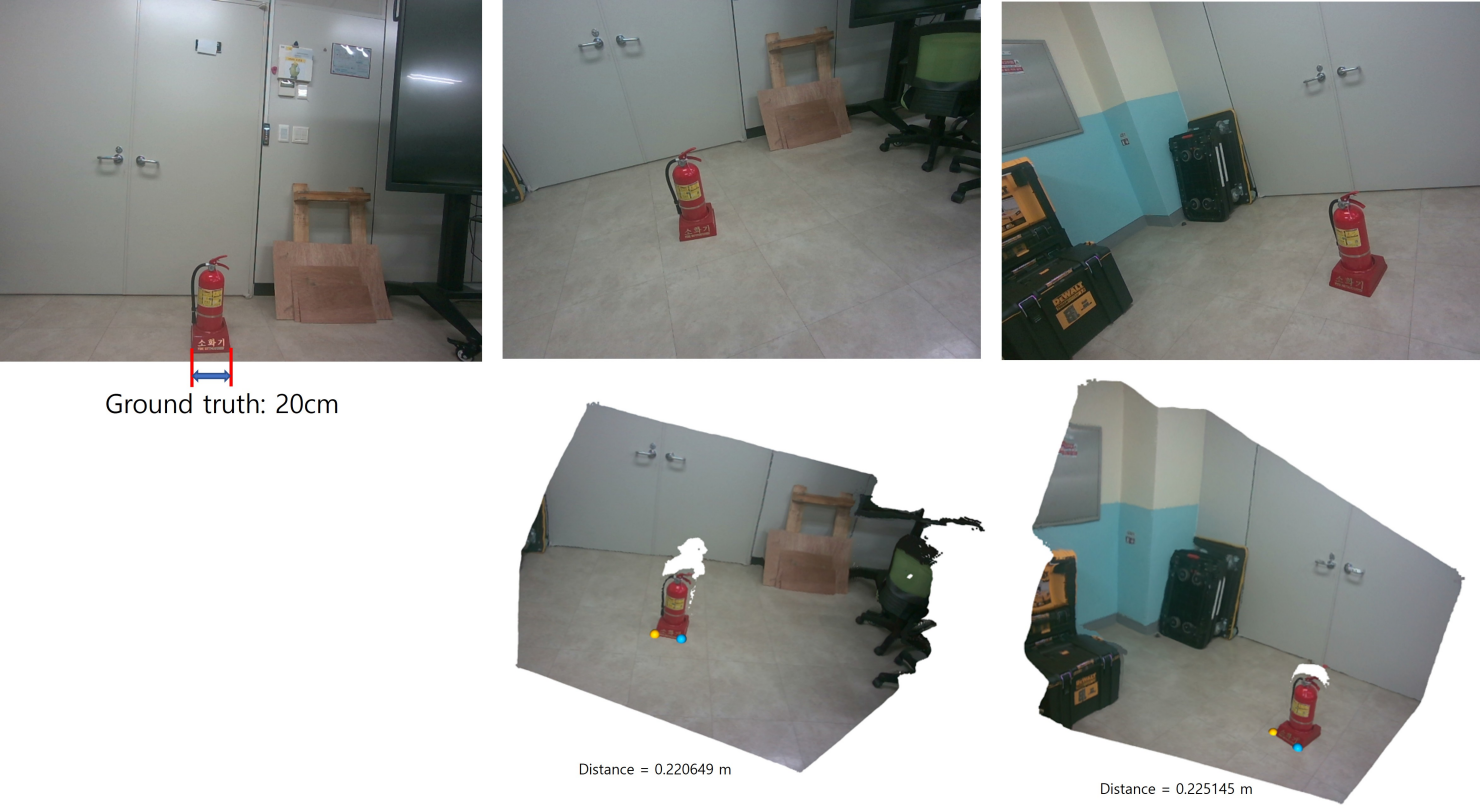
Viewpoint Variation Experiment with the MASt3R method [1]. The two images are captured with a translation of approximately 1.5 m and a viewing angle difference of 30°. Under these conditions, the estimated distances between the two endpoints of the fire extinguisher are 0.220649 m and 0.225145 m, respectively, corresponding to an absolute error of approximately 10% relative to the ground-truth distance (0.2 m). The difference between the two estimated distances is 0.004496 m, indicating high consistency in scale estimation across viewpoints.

Accurate estimation of the pose transformation requires that the two reconstructed point clouds share a consistent metric scale. In the proposed framework, MASt3R is employed to generate metrically scaled point maps. Therefore, the robustness of the estimated scale under large viewpoint and lighting variations largely depends on the robustness of MASt3R to these factors. Notably, MASt3R has been shown to be robust to significant viewpoint changes, particularly in the horizontal direction, due to its training on diverse wide-baseline data. To quantitatively evaluate the effect of viewpoint variation on scale consistency, we conducted an experiment as illustrated in Figure 4. The two images were captured with a translation of approximately 1.5 m and a viewing angle difference of about 30°. The ground-truth distance between the two endpoints of the fire extinguisher is 0.20 m. The corresponding distances estimated from the two reconstructed point clouds are 0.220649 m and 0.225145 m, respectively, which correspond to an absolute error of approximately 10% relative to the ground truth. Importantly, the difference between the two estimated distances is only 0.004496 m, indicating that the relative scale inconsistency between the two viewpoints is negligible. This suggests that, although there exists a moderate absolute scale error, the scale remains highly consistent across different viewpoints, which is sufficient for stable pose estimation in our framework. However, since MASt3R is a data-driven model, it may introduce relatively large metric errors when encountering conditions that are underrepresented or absent in the training data. For example, significant vertical viewpoint changes may lead to degraded scale accuracy.

**Figure 5:**
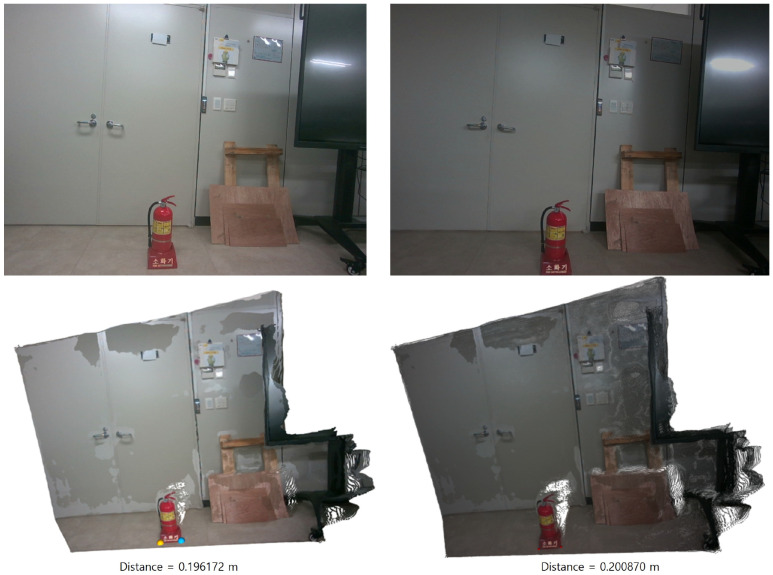
Lighting variation experiment with MASt3R method [1]. The two images are captured under different illumination conditions. Under these settings, the estimated distances between the two endpoints of the fire extinguisher are 0.196172 m and 0.200870 m, respectively. The difference between the two estimates is 0.004698 m, indicating high consistency in scale estimation across varying lighting conditions.

Regarding illumination variations, the authors of MASt3R report that the method is robust to day–night changes and describe it as “moderately robust” to general illumination variations. In particular, on the Aachen Day–Night benchmark, MASt3R demonstrates improved performance over DUSt3R, especially under nighttime conditions, with additional gains achieved through coarse-to-fine matching. In Figure 5, we demonstrate the effect of illumination changes on metric scale estimation. Under bright illumination, the estimated distance is 0.196172 m, while under dark illumination it is 0.200870 m. These values are closer to the ground-truth distance compared to the case with significant viewpoint variation, indicating that illumination changes have a smaller impact on scale accuracy than viewpoint changes. Furthermore, the difference between the two estimated distances is only 0.004698 m, suggesting that the metric scale remains highly consistent across different illumination conditions. Nevertheless, as noted by the authors of MASt3R, performance may degrade under more extreme appearance variations, such as severe seasonal changes or conditions that fall outside the training distribution.

#### 3.7.4. Limitations Under Partial Occlusion Conditions

Limited overlap between point clouds can degrade the stability of alignment and pose recovery. However, our framework mitigates this issue through the use of MASt3R, which is specifically designed to handle wide-baseline matching scenarios. Unlike traditional feature-based methods that rely on local keypoint overlap, MASt3R leverages dense, geometry-aware correspondences inferred from global context, enabling robust matching even under significant viewpoint variations. Using this robust matching, we have correspondence-informed ICP instead of the conventional ICP which re-estimates the correspondences between the points at every iteration using nearest-neighbor search. This allows the reconstruction of object geometry even when the overlap between observations is limited. Therefore, in practice, ICP can still converge to a correct solution when provided with a sufficiently informative subset of overlapping geometry. However, in extreme cases of minimal overlap, the accuracy of ICP-based alignment may degrade. In this case incorporating more advanced partial-overlap registration techniques like robust or weighted ICP variants is a promising direction for future work.

## 4. Experimental Results

In this section, we present experimental results on real-world photographs to demonstrate the effectiveness of the proposed method. We conducted experiments on ten real-world image pairs captured by us, as no publicly available dataset provides multi-view images of objects both before and after movement. Moreover, to the best of our knowledge, there is no existing method that estimates 3D object transformations from two-view images acquired before and after object movement. Consequently, direct quantitative comparisons with existing algorithms are not feasible.

**Figure 6:**
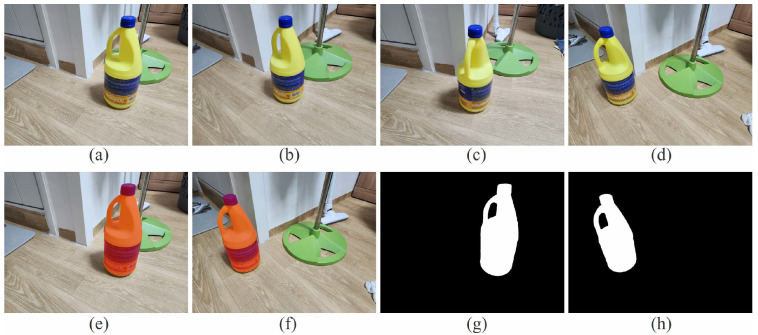
Representative example using real-world photographs. (**a**) First view before object movement, $I^{1,1}$; (**b**) second view before movement, $I^{1,2}$; (**c**) first view after movement, $I^{2,1}$; (**d**) second view after movement, $I^{2,2}$; (**e**,**f**) segmented object regions highlighted in red; (**g**,**h**) corresponding masked object regions.

Figure 6 presents results from one representative example. Figure 6a,b and Figure 6c,d depict two-shot images captured at different camera poses before and after the object’s movement, respectively. These images correspond to $I^{1,1}$, $I^{1,2}$, $I^{2,1}$, and $I^{2,2}$ as defined in the previous sections, and are used as inputs to the MASt3R network to generate the required 3D point maps. Figure 6e,f show the segmented object regions highlighted in red, while Figure 6g,h show the corresponding masked regions.

**Figure 7:**
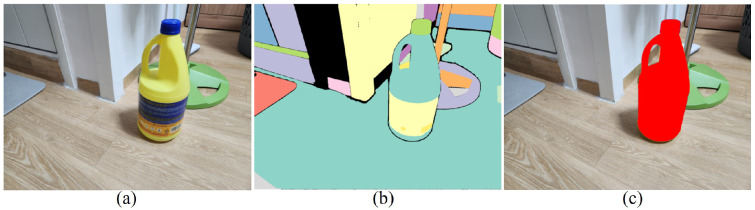
Procedure for extracting object regions from 2D images. (**a**) Input 2D image. (**b**) Multiple segmented regions obtained by applying the Segment Anything Model (SAM) [20]. (**c**) Final object region obtained by merging the masked regions located near the image center.

Figure 7 illustrates the procedure used to extract object regions from 2D images in our experiments. In our experiments, we adopt a basic 2D object region extraction method, and therefore, restrict evaluation to images for which this method successfully extracts object regions. We first applied the SAM (Segment Anything Model) [23] to the images to obtain multiple segmented regions, and then merged the segmented regions near the center to obtain the object region. However, we emphasize that this simple 2D object region extraction is not a contribution of our work; rather, our method is independent of the specific 2D extraction technique employed. Consequently, more advanced or robust 2D object region extraction methods can be readily integrated with the proposed 3D alignment framework when the simple approach used here is insufficient.

**Figure 8:**
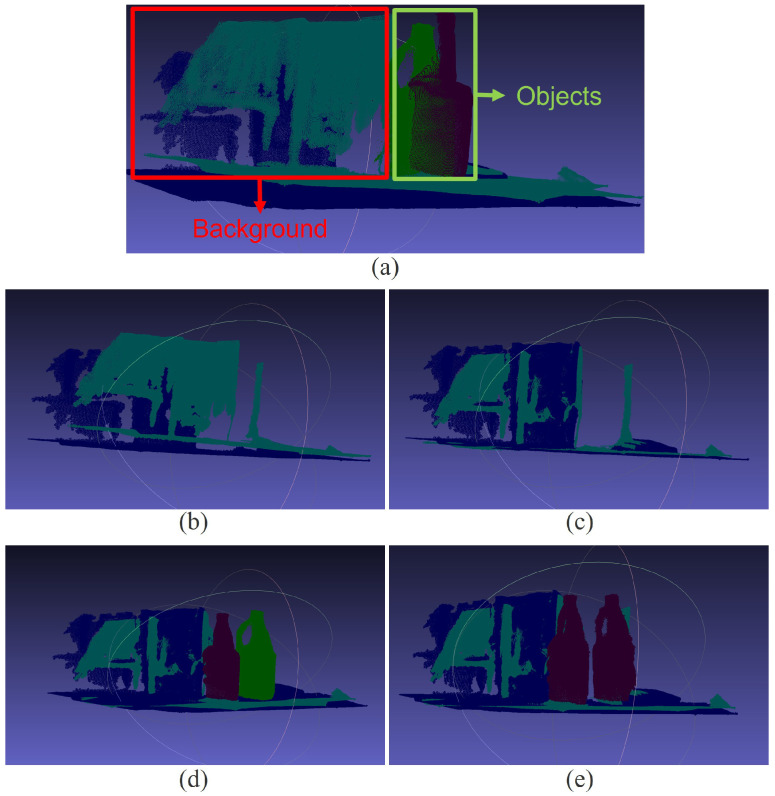
3D point cloud alignment results. (**a**) Overlaid point clouds from the reference and current days. (**b**) Misaligned background point clouds. (**c**) Aligned background point clouds after background registration. (**d**) Foreground and background point clouds transformed into a common reference coordinate system, where object point clouds remain non-overlapping due to object motion. (**e**) Final alignment of the original object point cloud to the moved object. The purple point cloud completely overlaps the green point cloud, indicating successful alignment.

After obtaining the object (foreground) region and the background region—where the background region is defined as the complement of the foreground within the entire image—we feed the 2D images into the MASt3R network to generate 3D point clouds. Using the foreground–background masks, the resulting 3D point clouds can then be separated into foreground and background components. Figure 8a shows the 3D point clouds corresponding to the 2D images in Figure 6, where the point clouds from the reference day and the current day are overlaid, and the foreground and background point clouds are separated.

Figure 8b shows only the background point clouds from the reference day and the current day, which are initially misaligned. Using Equation (7), the transformation parameters $R^{2\to 1}$ and $t^{2\to 1}$ are estimated; in our implementation, these parameters are obtained using the ICP method [19]. Applying $R^{2\to 1}$ and $t^{2\to 1}$, both the background and foreground point clouds are transformed into a common reference coordinate system. Figure 8c shows the aligned background point clouds, while Figure 8d presents the aligned background together with the aligned object point clouds, where object alignment follows Equation (10). Note that, due to the object motion, the object point clouds remain non-overlapping even after alignment to the same reference coordinate system. With the point clouds of the object before and after movement expressed in a common reference coordinate system, the transformation between the original and moved object can be estimated using Equation (11). Figure 8e illustrates original (unmoved) object point cloud overlaid onto the moved object point cloud using the estimated transformation parameters $R^{obj}$ and $t^{obj}$.

**Figure 9:**
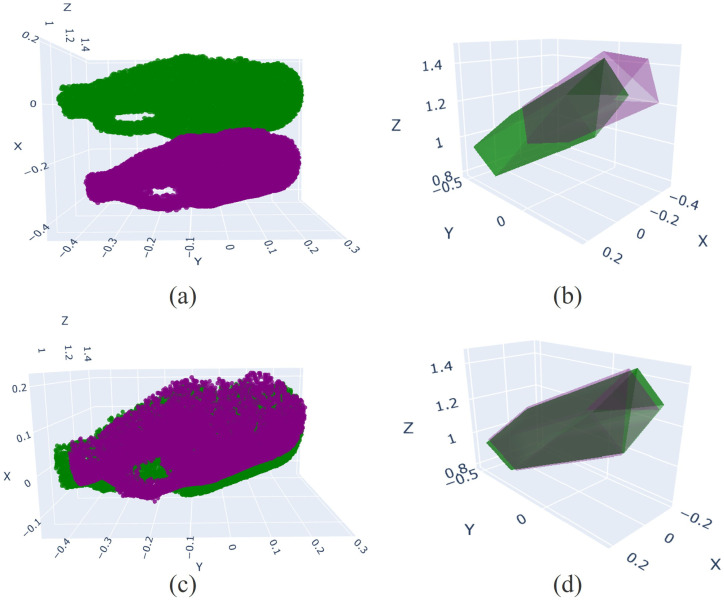
Simulation results of object alignment. (**a**) Object point clouds before alignment. (**b**) Bounding boxes of the non-aligned objects. (**c**) Object point clouds after alignment using the estimated transformation parameters. (**d**) Bounding boxes of the aligned objects. This visualization demonstrates that virtual objects corresponding to real-world objects can be accurately aligned using transformations estimated from real-world images.

Figure 9 again illustrates a simulation of aligning the original object with the moved object. Figure 9a and Figure 9c show the object point clouds before and after alignment, respectively, while Figure 9b,d present the corresponding bounding boxes. This visualization demonstrates that, in the virtual space, virtual objects corresponding to real-world objects can be accurately aligned using the transformation parameters estimated from real-world images.

**Figure 10:**
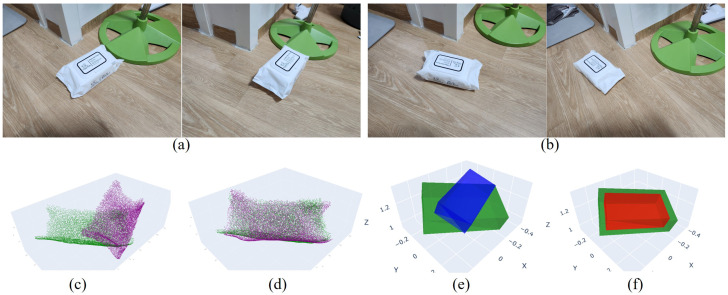
Results of object alignment. (**a**) Two views of the object before movement. (**b**) Two views of the object after movement. (**c**) Point clouds of the unmoved and moved objects prior to alignment. (**d**) Point clouds after alignment. (**e**) Bounding boxes prior to alignment. (**f**) Bounding boxes after alignment.

**Figure 11:**
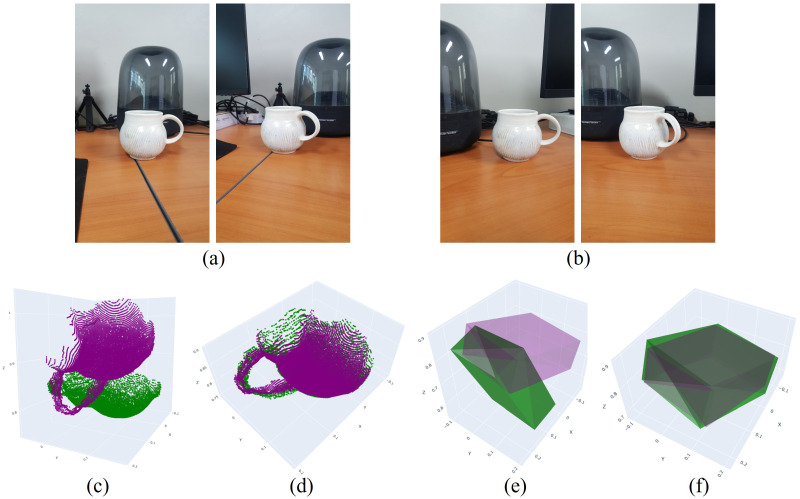
Results of object alignment. (**a**) Two views of the object before movement. (**b**) Two views of the object after movement. (**c**) Point clouds of the unmoved and moved objects prior to alignment. (**d**) Point clouds after alignment. (**e**) Bounding boxes prior to alignment. (**f**) Bounding boxes after alignment.

Figure 10 and Figure 11 present results for two additional objects. As in previous examples, the estimated transformations accurately align the objects and their corresponding bounding boxes to the moved position and pose. To quantitatively evaluate the proposed method, we employ two metrics: (i) the volumetric IoU (Intersection over Union) of the two 3D bounding boxes and (ii) the angular difference between their principal orientations, represented by the corresponding rotation matrices. In our evaluation, the 3D bounding box of each object is derived directly from the reconstructed point cloud by analyzing its spatial distribution. Specifically, the principal directions of the bounding box are determined based on the spread of the point cloud, where the longest axis corresponds to the direction of maximum variance (largest spatial extent), and the shortest axis corresponds to the direction of minimum variance. The bounding box is then defined to tightly enclose the point cloud along these axes. For IoU computation, we apply a centroid-based translation normalization, where the centers of the predicted and reference bounding boxes are aligned prior to overlap calculation. This translation normalization decouples translation error from shape and orientation evaluation. As a result, the reported IoU primarily reflects the consistency of the estimated object geometry and orientation, rather than being dominated by global translation offsets.

Table 1 reports the IoU and angular difference values for ten objects. It can be observed that, after aligning the 3D bounding boxes using the transformation parameters estimated by the proposed method, the evaluation metrics improve. For IoU estimation, the IoU between the moved and unmoved objects is typically zero due to their spatial separation. Therefore, we estimate the IoU after applying a translation that aligns the centroids (centers of mass) of the moved and unmoved objects.

## 5. Conclusions

This paper presented a background-aligned pose recovery framework for restoring real–virtual consistency in mixed-reality systems affected by intermittent tracking. By leveraging metrically scaled 3D reconstructions from MASt3R and estimating a global transformation from static background geometry, the proposed method reliably recovers object pose changes without requiring continuous tracking or prior motion information. Experiments on real-world scenes demonstrate that the proposed approach effectively aligns both object point clouds and their corresponding 3D bounding boxes under significant displacement and rotation, yielding substantial improvements in IoU and orientation accuracy. Future work will focus on extending the framework to more complex environments with multiple moving objects, integrating more robust 2D segmentation techniques, and enabling real-time operation in persistent mixed-reality systems. We believe this work represents a practical step toward long-term, reliable mixed-reality experiences.

## Figures and Tables

**Table 1:** Orientation error and IoU for the evaluated images. The unaligned IoU is computed after applying a translation that aligns the centroids of the moved and unmoved objects.

Image Number	Original Orientation Error (°)	Aligned Orientation Error (°)	Unaligned IoU	Aligned IoU
Image1	54.02	5.05	0.503	0.953
Image2	30.44	7.21	0.123	0.926
Image3	65.54	11.62	0.451	0.815
Image4	73.21	8.24	0.391	0.921
Image5	54.33	5.66	0.125	0.876
Image6	44.82	6.43	0.305	0.954
Image7	26.37	7.27	0.528	0.963
Image8	31.23	3.42	0.439	0.994
Image9	52.81	9.71	0.463	0.912
Image10	28.73	7.58	0.217	0.925

## Data Availability

The original contributions presented in this study are included in the article. Further inquiries can be directed to the corresponding author.

## Nomenclature

MASt3R	Matching and Stereo 3D Reconstruction
DUSt3R	Dense Unconstrained Stereo 3D Reconstruction
AR	Augmented Reality
VR	Virtual Reality
MR	Mixed Reality
SLAM	Simultaneous Localization and Mapping
ICP	Iterative Closest Point
GPU	Graphics Processing Unit
SAM	Segment Anything Model
IoU	Intersection over Union
